# Signs of left atrial disease and 10-year risk of atrial fibrillation

**DOI:** 10.1371/journal.pone.0266848

**Published:** 2022-04-22

**Authors:** Tobias Uhe, Tina Stegmann, Romy Langhammer, Nikolaos Dagres, Ulrich Laufs, Rolf Wachter

**Affiliations:** 1 Klinik und Poliklinik für Kardiologie, Universitätsklinikum Leipzig, Leipzig, Germany; 2 Abteilung für Rhythmologie, Herzzentrum Leipzig, Leipzig, Germany; Ohio State University, UNITED STATES

## Abstract

**Background:**

The contribution of left atrial disease and excessive supraventricular ectopic activity (ESVEA) to the risk for incident atrial fibrillation (AF) is incompletely understood.

**Objective:**

To analyse the ten-year risk to develop AF in patients with cardiovascular risk factors and to define the impact of parameters of left atrial disease and ESVEA on AF risk.

**Methods:**

148 patients from the Diast-CHF trial with at least one cardiovascular risk factor and free of AF at baseline were followed for 10 years. Left atrial disease was defined as left atrial volume index (LAVI) >35 ml/m^2^, P-terminal force in lead V_1_ (PTFV_1_) >4000 ms*μV or elevated N-terminal pro-B-type natriuretic peptide (NT-proBNP) >250 pg/ml. We analyzed the association of these parameters and ESVEA (either >720 premature atrial contractions (PAC) or one atrial run >20 beats per day) on AF-free survival.

**Results:**

After ten years, AF was newly detected in twelve patients (13.4%) with signs of left atrial disease and two patients (3.4%) without signs of left atrial disease (p = 0.04). LAVI (p = 0.005), ESVEA (p = 0.016) and NT-proBNP (p = 0.010) were significantly associated with AF-free survival in univariate analysis. A combined Cox model of left atrial disease parameters showed associations for NT-proBNP (HR 3.56; 95%CI 1.33–5.31; p = 0.04) and PAC (HR 2.66; 95%CI 1.25–10.15; p = 0.01) but not for LAVI or PTFV_1_ with AF-free survival.

**Conclusion:**

The risk for AF is higher in patients with cardiovascular risk factors and signs of left atrial disease. NT-proBNP and premature atrial contractions independently predict AF-free survival. The role of excessive supraventricular ectopic activity for the assessment of AF risk may be underestimated and requires further study.

## 1. Introduction

Atrial fibrillation (AF) is the most common rhythm disorder and one of the major risk factors for ischemic stroke. Several risk factors for AF have been identified and recently, the concept of left atrial cardiomyopathy (LACMP) has been developed and proposed to play a key role in the development and progression of AF [1]. However, the definition of LACMP or left atrial disease is a matter of discussion and the impact of its components on AF risk has not been studied in detail. The risk of AF is increased with age and cardiovascular comorbidities [2]. Biomarkers reflecting left atrial abnormalities, e.g., enlargement or fibrosis have been proposed and shown to improve AF risk stratification. The most established biomarkers are echocardiographic and electrocardiographic indicators for enlargement of the left atrium and natriuretic peptides [3].

Echocardiographic left atrial enlargement measured in one dimension has been shown to be a predictor of AF in the Framingham Heart Study and Cardiovascular Health Study [4, 5]. Moreover, Tsang et al. proposed the left atrial volume to be a better predictor for AF than one-dimensional parameters due to its higher accuracy [6]. P-terminal force in V_1_ (PTFV_1_) is considered as an electrocardiogram (ECG) indicator of left atrial enlargement and was associated with AF risk in multiple studies [7]. Elevated natriuretic peptides were predictive of death and cardiovascular events in the Framingham Heart Study [8]. Furthermore, they have been shown to be an independent risk factor for AF in the Cardiovascular Health Study [9].

The current concept of a left atrial disease does not include excessive supraventricular ectopic activity (ESVEA), although ESVEA increased the risk for AF in the population-based Copenhagen Holter Study, in patients with cardiovascular risk factors and patients post stroke [10–13].

We therefore hypothesized that not all of the currently described components associated with a left atrial disease do have an independent impact on AF risk and that ESVEA is a major independent risk factor for AF development.

## 2. Methods

### Patients

This analysis was conducted in a subgroup of participants of the non-interventional Diast-CHF (Prevalence and clinical course of diastolic dysfunction and diastolic heart failure) trial. The design, inclusion and exclusion criteria of the Diast-CHF trial have been described previously [14]. In summary, patients with at least one cardiovascular risk factor for heart failure with preserved ejection fraction aged 50 to 85 years were included in the Diast-CHF trial. The patients had at least one of the following risk factors: hypertension, diabetes mellitus, sleep apnoea syndrome or atherosclerotic disease. Patients who participated in the 7-day Holter ECG substudy (n = 162) qualified for the current analysis [14]. All participants underwent medical history, physical examination, a 12-lead ECG and transthoracic echocardiography at baseline. Follow-up investigations were conducted after one, two, five, nine and ten years. Patients with AF in medical history or in 12-lead- or Holter-ECG at baseline were excluded from further analyses. The protocol of the Diast-CHF study was approved by the responsible ethics committee of University Medicine Göttingen and complies with the Declaration of Helsinki. All patients gave their informed written consent.

### N-terminal pro-B-type natriuretic peptide (NT-proBNP)

NT-proBNP plasma levels were measured using a sandwich immunoassay (Roche Diagnostics, Mannheim, Germany). For the diagnosis of a left atrial disease, NT-proBNP > 250 pg/ml was considered abnormal [15]. For multivariable analysis, we used log-transformed NT-proBNP.

### 12-lead ECG

12-lead ECGs were obtained using ECG machines calibrated at 10 mm/mV with a speed of 50 mm/s. PTFV_1_ was measured as described previously in two consecutive p-waves and mean PTFV_1_ was calculated [16]. PTFV_1_ >4000 ms*μV was defined as abnormal according to previously published data [17].

### Echocardiography

Echocardiography was performed by experienced physicians using a Sonos 5500 (Hewlett-Packard, Andover, MA, USA) according to the guidelines of the American Society of Echocardiography and as previously described [18]. Indexed left atrial volume (LAVI) was calculated using left atrial volume and body surface area [19].

### Holter ECG

A dual-channel Holter ECG was recorded with digital portable recorders (Lifecard CF, Del Mar Reynolds Medical Ltd, Hertford, UK) for seven consecutive days. Analyses were performed with Pathfinder digital (Software Version V8.602, Del Mar Reynolds Medical Ltd). Holter-ECG were systematically screened for AF by an experienced investigator. Excessive supraventricular ectopic activity was defined as >720 premature atrial contractions (PAC) or at least one atrial run > 20 beats per day according to data from the Copenhagen Holter study and used as a categorical variable for survival analysis [10]. For multivariable analysis, we used log-transformed PAC.

### Definition of left atrial disease

Left atrial disease was defined as described previously as either PTFV1 > 4000 ms*μV, NT-proBNP > 250 pg/ml or LAVI > 35 ml/m^2^ [15].

### Endpoint assessment

Patients received ECGs at the regular study visits. If patients reported AF episodes outside of the study visits, original documentation (e.g., ECG tracings, hospital letters) was requested and critically reviewed. AF was defined according to current guidelines as any episode of at least 30 seconds during Holter ECG monitoring or documentation on a 12-channel ECG strip [20]. An experienced cardiologist, blinded to all other clinical data, validated all AF episodes.

### Statistical analysis

Continuous variables are given as mean ± standard deviation if normally distributed and as median and interquartile ranges between the 25^th^ and 75^th^ centile if skew distributed. Categorical variables are shown as absolute numbers (%). Continuous data were compared by Student’s t-test, skew distributed data by Mann-Whitney-U-Test and frequencies by Fisher’s exact test or chi-square test.

AF free survival was calculated using Kaplan-Meier plots with log-rank test for PTFV_1_ > 4000 ms*μV, NT-proBNP > 250 pg/ml, LAVI > 35 ml/m^2^ and ESVEA. Thereafter, multivariable analysis using Cox-regression was performed for all parameters associated with AF free survival and possible confounders, i.e., parameters with significant differences in baseline characteristics. Skew distributed parameters were log-transformed.

All tests were performed with SPSS Statistics 25.0 (IBM, Chicago, Illinois, USA). P-values < 0.05 were considered to be significant.

## 3. Results

### Study population

**Fig 1:**
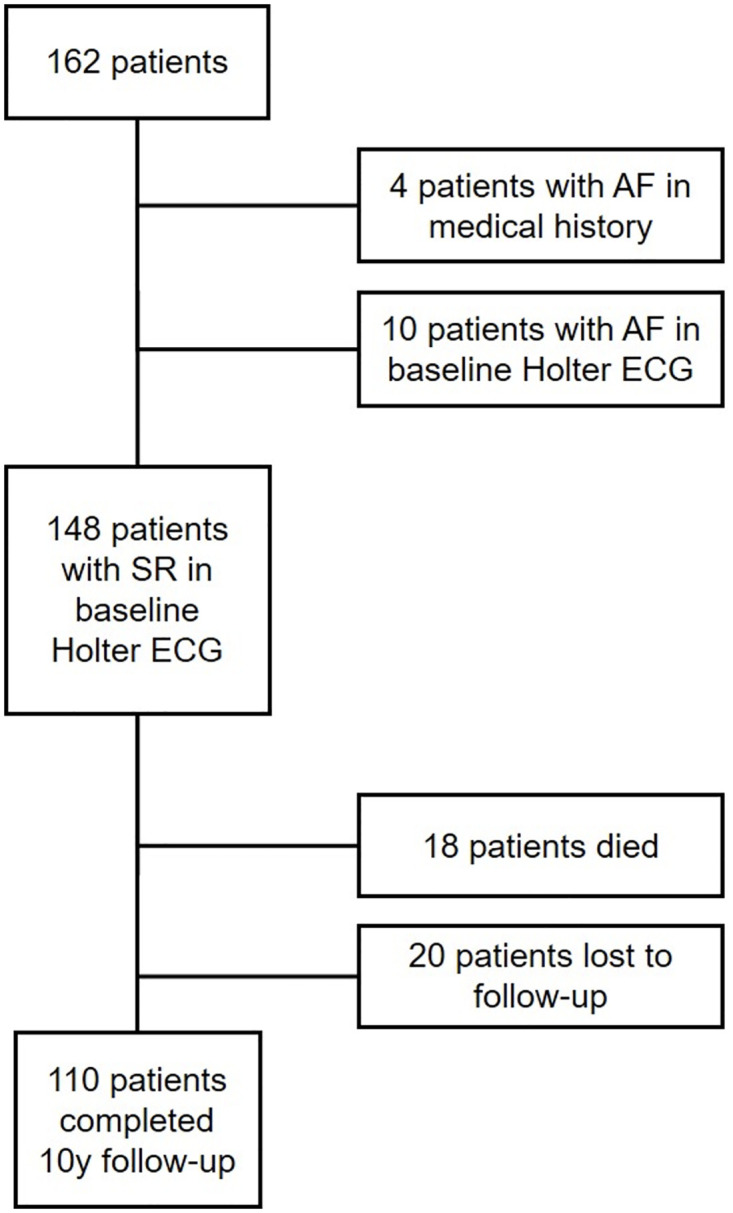
Study flow chart. AF = Atrial fibrillation; SR = Sinus rhythm; Of 162 patients described in Seegers et al. [14], 4 patients had AF in medical history and 10 had AF in baseline Holter ECG. Of 148 patients with SR in Holter ECG, 110 patients completed 10y-follow-up, 18 patients died, and 20 patients were lost to follow-up for other reasons.

Of the 162 patients with evaluable Holter ECG, four patients had AF in medical history and ten patients in baseline Holter. 148 were included in the present analysis. 110 patients completed follow-up after 10 years while 18 patients died, and 20 patients were lost to follow-up. The study flow chart is shown in Fig 1.

### Distribution of parameters of left atrial disease and baseline characteristics

**Fig 2:**
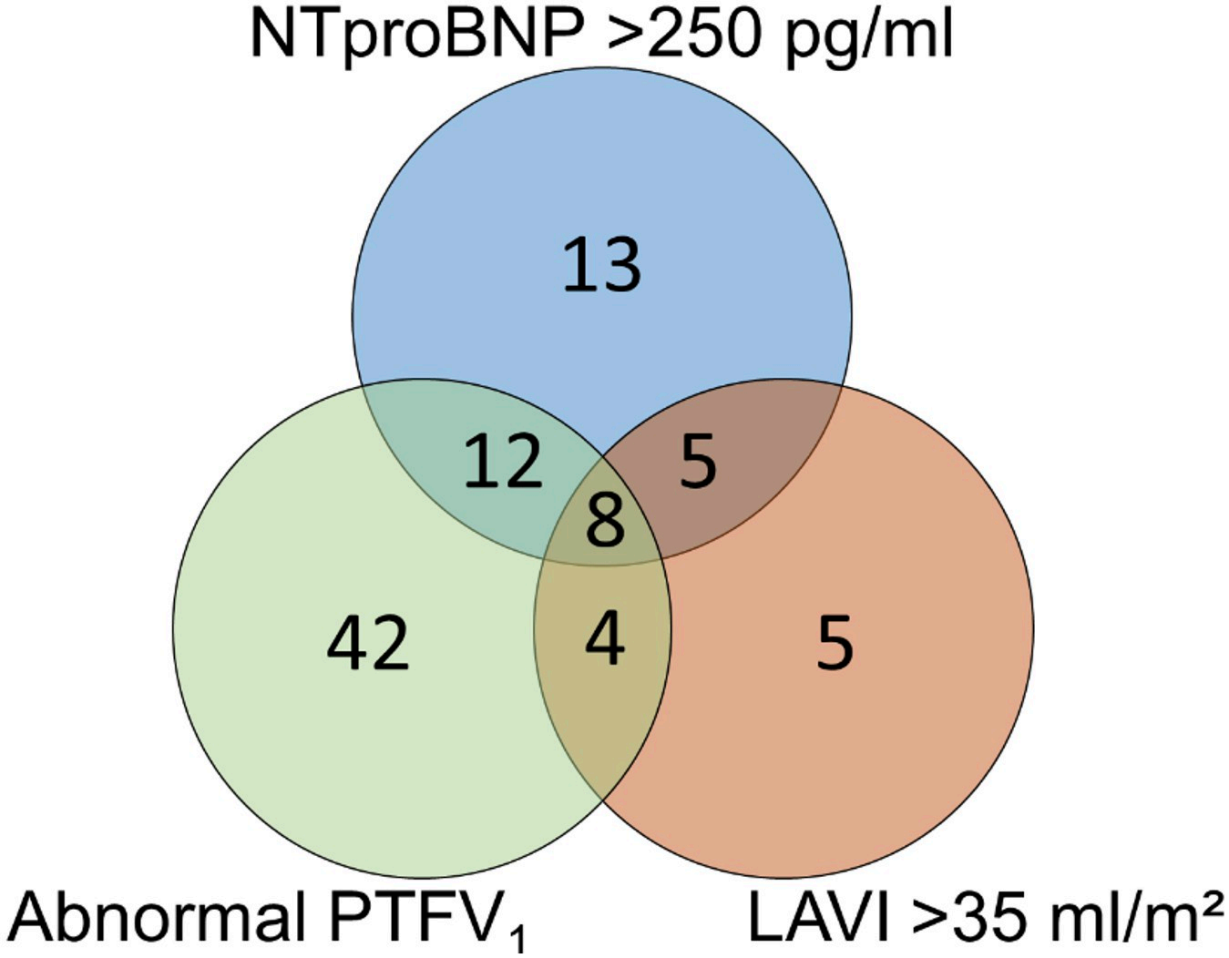
Venn diagram of distribution of left atrial disease parameters. 66 patients (45%) had abnormal PTFV_1_ (green circle), 38 patients (26%) had elevated plasma levels of NTproBNP (blue circle), and 22 patients (15%) had elevated left atrial volume index (red circle). LAVI = indexed left atrial volume; PTFV_1_ = P-terminal force in V_1_, NT-proBNP = N-terminal pro-B-type natriuretic peptide.

Eighty-nine patients had at least one parameter of left atrial disease while eight patients fulfilled all criteria. Sixty-six patients (45%) had abnormal PTFV_1_, 38 patients (26%) had elevated NT-proBNP, 22 patients (15%) had LAVI > 35 ml/m^2^ (Fig 2).

Table 1 shows the baseline characteristics of patients with at least one parameter of left atrial disease compared to those without. Patients with signs of left atrial disease were significantly older, had a higher prevalence of coronary artery disease, higher systolic blood pressure at baseline and a prolonged PQ-interval. No significant differences were found for indexed left ventricular mass and echocardiographic diastolic dysfunction measured as E/e’.

**Table 1:** Baseline characteristics for patients with and without signs of left atrial disease.

	No signs of left atrial disease (n = 59)	Signs of left atrial disease (n = 89)	p
Age (years)	62.0 ± 7.2	65.5 ± 7.3	0.01*
Male gender	33 (55.9)	54 (60.7)	0.57
Systolic blood pressure (mmHg)	143.3 ± 18.8	150.6 ± 20.4	0.03*
Diastolic blood pressure (mmHg)	84.1 ± 12.0	84.4 ± 12.2	0.91
Heart rate (/min)	74.1 ± 14.0	70.9 ± 13.9	0.17
BMI (kg/m^2^)	29.2 ± 5.1	28.7 ± 4.0	0.55
Comorbidities			
Hypertension	54 (91.5)	82 (92.1)	1.00
Heart failure	4 (6.8)	5 (5.6)	1.00
Diabetes mellitus	18 (30.5)	25 (28.1)	0.75
Hyperlipidaemia	25 (42.4)	44 (49.4)	0.40
Sleep apnea	3 (5.1)	7 (7.9)	0.74
Coronary artery disease	7 (11.9)	24 (27.0)	0.03*
Myocardial infarction	5 (8.5)	12 (13.5)	0.35
Periphery artery disease	3 (5.1)	2 (2.2)	0.39
Stroke/TIA	4 (6.8)	1 (1.1)	0.08
CHA_2_DS_2_-VASc Score	2 [1;3]	3 [2;3]	0.21
Medication			
Beta-blocker	26 (44.1)	41 (46.1)	0.81
AT1-Antagonists	12 (20.3)	10 (11.2)	0.13
ACE-Inhibitors	29 (49.2)	45 (50.6)	0.87
Thiazide diuretics	29 (49.2)	41 (46.1)	0.71
Loop diuretics	5 (8.5)	15 (16.9)	0.14
MRA	1 (1.7)	0 (0)	0.40
CCB	8 (13.6)	20 (22.5)	0.18
Laboratory			
Creatinine (mg/dl)	.96 ± .17	1.02 ± .35	0.22
Hemoglobine (g/dl)	14.38 ± 1.17	14.10 ± 1.20	0.18
TSH (μU/ml)	1.14 ± .71	1.03 ± 1.19	0.51
12-lead-ECG			
PQ (ms)	162.2 ± 20.4	171.4 ± 28.9	0.03*
QRS (ms)	91.4 ± 12.9	92.3 ± 13.3	0.68
QT interval (ms)	382.4 ± 27.5	392.2 ± 36.1	0.07
QTc Bazett (ms)	402.5 ± 23.3	403.6 ± 26.3	0.79
Echocardiography			
LVEF (%)	60.4 ± 5.9	60.2 ± 6.7	0.84
Left atrial diameter (mm)	39.4 ± 4.3	42.0 ± 5.6	0.01*
A’ (m/s)	10.2 ± 2.1	10.0 ± 2.3	0.70
Left ventricular mass index (g/m^2^)	75.8 ± 30.8	84.2 ± 38.3	0.16
E/e’ >14	2 (3.4%)	10 (11.2%)	0.09

Baseline characteristics; n = 148; left atrial disease is defined as either LAVI > 35 ml/m^2^ or PTFV1 ≤ -4000 ms*μV or NT-proBNP > 250 pg/ml); BMI = Body mass index; TIA = transient ischaemic attack; PTFV_1_ = P-terminal force in V_1_; NT-proBNP = N-terminal pro-B-type natriuretic peptide; MRA = mineralocorticoid receptor antagonists; CCB = calcium-channel blockers; TSH = Thyroid-stimulating hormone; ECG = electrocardiogram; LVEF = Left ventricular ejection fraction; Values are given as mean ± standard deviation, median [interquartile range] or n (%)

### Risk of atrial fibrillation in 10-year-follow-up

**Fig 3:**
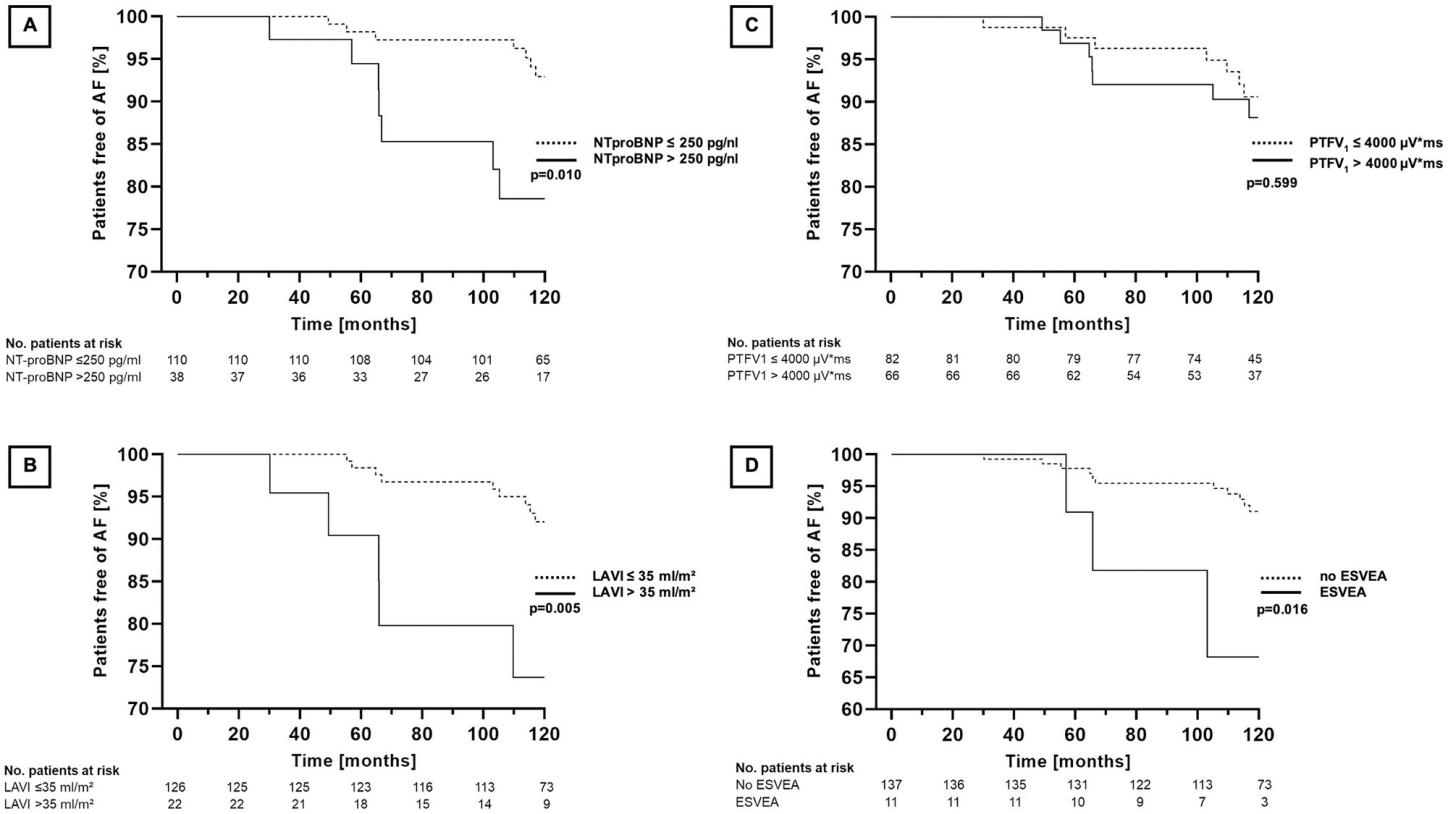
Kaplan-Meier curves of AF-free survival for the different parameters of left atrial disease. (A) AF-free survival in patients with NT-proBNP ≤250 pg/ml and >250 pg/ml. (B) AF-free survival in patients with LAVI ≤35 ml/m^2^ and >35 ml/m^2^. (C) AF-free survival in patients with PTFV1> 4000μV*ms and PTFV1≤4000μV*ms. (D) AF-free survival in patients with ESVEA and no ESVEA. AF = Atrial fibrillation; LAVI = Left atrial volume index; ESVEA = Excessive supraventricular ectopic activity.

After ten years of follow-up, AF was newly detected in twelve patients (13.4%) with signs of left atrial disease and two patients (3.4%) without signs of left atrial disease (p = 0.04). In univariate analysis, NT-proBNP level ≤ 250 pg/ml (p = 0.01) and LAVI ≤ 35 ml/m^2^ (p = 0.01), showed a significant association with AF free survival, while PTFV_1_ did not (p = 0.60). ESVEA was significantly associated with a reduction in AF-free survival (p = 0.02). Kaplan-Meier-plots are shown in Fig 3.

In multivariable analysis, significant associations with AF-free survival were found for log-transformed NT-proBNP and log-transformed PAC, but not LAVI (see Table 2).

**Table 2:** Multivariable analysis of revised parameters of left atrial disease on AF-free survival.

Parameter	Hazard Ratio	95% Confidence Interval		p-Value
logPAC	2.66	1.25	10.15	0.01
logNT-proBNP	3.56	1.33	5.31	0.04

NT-proBNP = N-terminal pro-B-type natriuretic peptide; PAC = premature atrial contractions.

## 4. Discussion

We studied the impact of parameters of a left atrial disease on the risk of atrial fibrillation in patients with cardiovascular risk factors. The main findings are that NT-proBNP and premature atrial contractions were independently associated with recurrent AF occurrence but not the left atrial volume index or the P-terminal force in lead V_1_.

Our finding of a predictive value of natriuretic peptides to identify patients at risk for paroxysmal AF is corroborated by findings in other cohorts, e. g. population-based and patients immediately post stroke [21, 22]. In Diast-CHF, we previously showed that natriuretic peptides are predictive for current paroxysmal AF and now extend these data on 10-year long-term risk of AF.

Left atrial enlargement has been shown to be predictive for AF in patients with cardiovascular risk factors, population based cohorts and patients post stroke [4, 5, 23]. Most studies investigated left atrial diameter (LAD), not left atrial volume, although LAVI has been recognized as a better predictor of AF than LAD or indexed LAD [6]. However, most studies performed multivariable analyses with echocardiographic measurements and clinical covariates but did not analyze ESVEA and PTFV_1_. We observed an association between incident AF and LAVI in univariable analysis, but this association disappeared after including ESVEA and NT-proBNP in a multivariable model. LAVI and NT-proBNP are often correlated and bare similar diagnostic information [24]. Our results imply that LAVI does not have incremental diagnostic value over NT-proBNP for the prediction of AF. Pagola et al. came to a similar conclusion following the comparative analysis of NT-proBNP and LAVI as risk predictors for AF in patients after cryptogenic stroke [25].

Our analysis did not find an impact of electrocardiographic abnormality, i.e., increased PTFV_1_ on AF-free survival. In contrast, several analyses have shown significant associations of PTFV_1_ with the risk of AF [17, 26]. This discrepancy is probably due to different patient characteristics of these north American cohort studies compared to our study, which included Caucasians only. PTFV_1_ was highly predictive of AF in black patients, but only moderately in white patients [26]. This race-dependent difference could be a possible explanation for the failure of PTFV_1_ as an AF risk predictor in our study. Moreover, the clinical utility of PTFV_1_ may be limited because of its poor inter-observer- and inter-P-waves-reliability [27].

Excessive supraventricular ectopic activity was associated with the 10-year risk for AF. Consistent with our findings, ESVEA was predictive of AF post stroke and in the population based Copenhagen Holter study [10, 12, 13].

An independent predictive value of ESVEA and NT-proBNP on AF risk was shown in a population-based Swedish cohort, but a direct comparison of four potential components of left atrial disease (NT-proBNP, left atrial size, PTFV_1_ and ESVEA) on the risk of AF development has not been performed yet [28]. From a methodological standpoint, it is suggestive that some components may bear similar diagnostic information. Our analysis found that PTFV_1_ and left atrial size do not give incremental predictive information on the risk of AF. In a multivariable analysis of data from the Framingham Heart Study (PTFV_1_ was not significantly associated with AF [29]. Rasmussen et al. evaluated different P-wave-indices from the Copenhagen Holter study and incorporated them into a clinical risk score developed from data of the Framingham Heart Study but could not show an improved AF risk discrimination for PTFV_1_ [30]. Adding echocardiographic measurements to a clinical AF risk score showed a slight impact on the risk assessment [31]. Interestingly, in 5120 participants in the Cardiovascular Health Study, left atrial dimension was the only parameter of left atrial disease parameter not significant associated with stroke risk–even if ESVEA was not included in the analysis [3].

Another interesting finding is that age did not give predictive information for AF. A potential explanation is that all four potential components of left atrial disease were positively correlated with age [32–35]. This may explain why age per se did not bear incremental diagnostic information.

## 5. Strengths and limitations

Our analysis is limited by a medium sample size and a moderate number of patients lost to follow-up, which reduces the power of our analysis. Moreover, in the Diast-CHF-study, cardiovascular imaging was achieved by transthoracic echocardiography, not by cardiac magnetic resonance imaging (CMR). This can be considered as a limitation as CMR has the potential to acquire more accurate data regarding the left atrial volume and to quantify left-atrial fibrosis. Our analysis is also limited by lacking a time-dependent analysis of the development of different parameters of left atrial disease. Still, the detailed and comprehensive characterization of the patient population is a strength of the analysis. The 7-day Holter ECG prior to study inclusion is minimizes the risk of the inclusion of patients with undetected underlying and easily detectable AF.

## 6. Conclusion

In summary, out of four potential predictors for incident atrial fibrillation, only the excessive supraventricular ectopic activity and NT-proBNP were independently associated with incident atrial fibrillation during a 10-year follow-up. ESVEA should be considered as a potential criterion for a left atrial disease. Further studies are wanted to validate these findings in different cohorts and prospectively.

## Supporting information

S1 Data (XLSX)

## Abbreviations

AF: atrial fibrillation
ECG: electrocardiogram
ESVEA: excessive supraventricular ectopic activity
LACMP: left atrial cardiomyopathy
LAD: left atrial diameter
LAVI: indexed left atrial volume
NT-proBNP: N-terminal pro-B-type natriuretic peptide
PAC: premature atrial contractions
PTFV_1_: P-terminal force in V_1_
